# Translog, a web browser for studying the expression divergence of homologous genes

**DOI:** 10.1186/1471-2105-11-S1-S59

**Published:** 2010-01-18

**Authors:** Xianjun Dong, Altuna Akalin, Yogita Sharma, Boris Lenhard

**Affiliations:** 1 Computational Biology Unit, Bergen Center for Computational Science, University of Bergen, Thormøhlensgate 55, N-5008 Bergen, Norway; 2 Sars Centre for Marine Molecular Biology, University of Bergen, Thormøhlensgate 55, N-5008 Bergen, Norway

## Abstract

**Background:**

Increasing amount of data from comparative genomics, and newly developed technologies producing accurate gene expression data facilitate the study of the expression divergence of homologous genes. Previous studies have individually highlighted factors that contribute to the expression divergence of duplicate genes, e.g. promoter changes, exon structure heterogeneity, asymmetric histone modifications and genomic neighborhood conservation. However, there is a lack of a tool to integrate multiple factors and visualize their variety among homologous genes in a straightforward way.

**Results:**

We introduce Translog (a web-based tool for Transcriptome comparison of homologous genes) that assists in the comparison of homologous genes by displaying the loci in three different views: promoter view for studying the sharing/turnover of transcription initiations, exon structure for displaying the exon-intron structure changes, and genomic neighborhood to show the macro-synteny conservation in a larger scale. CAGE data for transcription initiation are mapped for each transcript and can be used to study transcription turnover and expression changes. Alignment anchors between homologous loci can be used to define the precise homologous transcripts. We demonstrate how these views can be used to visualize the changes of homologous genes during evolution, particularly after the 2R and 3R whole genome duplication.

**Conclusion:**

We have developed a web-based tool for assisting in the transcriptome comparison of homologous genes, facilitating the study of expression divergence.

## Background

One of the challenges in the post-genomic era is to understand the mechanisms which drive the divergence of gene expression, and how this causes phenotypic changes, ultimately leading to the evolution of new species [1-6]. This is important both at the level of orthologs (genes separated by a speciation even) and paralogs (genes separated by a duplication event). For example, *PAX6*, the most studied Pax gene, is a "master control" gene for the development of eyes and sensory organs, and other homologous structures, usually derived from ectodermal tissues [7]. Its protein function is highly conserved across bilaterian species: mouse *PAX6 *can trigger eye development in *D. melanogaster *[8]. However, genomic organization of genes sharing the ancestry with the human *PAX6 *and its immediate neighborhood varies considerably among species, with differences in the number and distribution of exons, *cis*-regulatory elements and transcription start sites. For paralogous genes, derived from gene duplication or whole genome duplication, it has been shown that duplicate genes increase expression divergence and enable tissue or developmental specialization to evolve, as shown in mammals [9], fish [10], worm[10], yeast[11], and plants[12]. By comparing the transcription patterns of duplicate genes, we can often trace the factors that influence the expression pattern changes in evolution.

At the genomic level, previous studies have focused on examining the relationship between the divergence of gene expression and type of the promoter[13], exon structure[14], TSS turnover[14], genomic neighborhood[15], cis-regulatory inputs [16], histone modifications[17], and recently, the DNA-encoded nucleosome organization of promoters , possibly further complicated by external environmental factors are involved [18].

The increasing volume of available transcriptome data such as CAGE[19] and RNA-seq [20] for different developmental stages and tissues for different species can be harnessed to understand the mechanisms of spatiotemporal expression changes of genes that share a (not so ancient) common ancestor. The investigation should start with the integrated analysis of the available data. A suitable tool for this type of analysis should enable the comparison of homologous genes on different scales, from the position and activity of their proximal promoters to the corresponding information on their long-range regulatory inputs. Similar tools, like the comparative genome viewer in DBTSS[21], also contribute to compare the promoter and transcripts for homolog genes, but they don't use high-throughput sequencing like CAGE and their visualization methods are not so enhanced. In this paper, we describe Translog (the tool for **Trans**criptome comparison of homo**log**s) [22], a web-based application providing 1) a *promoter *view where a region containing all proximal promoters of a gene's transcript(s) is aligned to its homolog and cross-mapped between the two loci using alignment anchors, 2) a *gene structure *view where a gene's exon-intron structure is compared to that of its homolog, alongside its transcriptional features, and 3) a *genomic neighborhood *view which displays the neighbors of a gene in a large flanking region, and show their conservation in the homologous loci. CAGE data is displayed along with the genomic features to indicate the expression of transcripts. We demonstrate how Translog can be used to discover and visualize homologous relationships, expression pattern changes after duplication or speciation, and to explore the divergences of promoter usage, gene structure and genomic neighborhood between two homologs. We anticipate that Translog will be useful in looking for the factors of impacting expression divergence between two homologous genes, and finally contribute to understanding the mechanism of evolution of gene expression.

## Methods

### Gen(om)e annotations

To define the promoter region and gene structure, we use the gene name and genomic locations of all Ensembl genes and transcripts from Ensembl v52 [23]. Currently, these include three genomes (human, mouse and zebrafish): 25233 genes in *D. rerio *(assembly version 7), 37436 in *H. sapiens *(assembly NCBI Build 36.1), and 31805 in *M. musculus *(assembly NCBI Build 37). We use these three species because i) there is CAGE data available for them, and ii) comparison of human:human, zebrafish:zebrafish paralogs can reveal the expression changes along with 1R/2R, 3R whole genome duplication, respectively. The orthologs (human:mouse, human:zebrafish, human:tetraodon) and paralogs (human:human) were downloaded from Ensembl Compara v52 [23], using BioMart[24]. For zebrafish:zebrafish paralogs, instead of taking all paralogs from Ensembl, we are primarily interested in those duplicates arisen in the event of fish-specific WGD. For the latter, we used human:zebrafish orthologs as a bridge (approximating ancestral genome before WGD) to extract those zebrafish gene pairs which have the same human ortholog genes. RefSeq genes were downloaded from refGene table in UCSC Table Browser (on 2009-08-01) for each genome.

### Defining TSS using CAGE tag clusters

In order to define CAGE TSSes and clusters, we used all publicly available CAGE tags (from http://fantom.gsc.riken.jp/4/download/, [25]) for human (hg18) and mouse (mm9). We used only uniquely mapping tags and clustered CAGE tags into tag clusters (TCs) if the member tags map to the same chromosome strand and overlap by at least 1 bp. For each TC we defined a representative location (as that supported by the highest number of tags). Afterwards, we grouped TCs into Sharp or Broad promoters using previous classification algorithm [26]. TCs are mapped to Ensembl genes on the [-500 bp, +500 bp] region around Ensembl TSS. If multiple TCs map to a given region, the one with the highest number of tags per million (tpm) is selected as representative TC for the gene.

### Alignments of homologous loci

**Figure 1:**
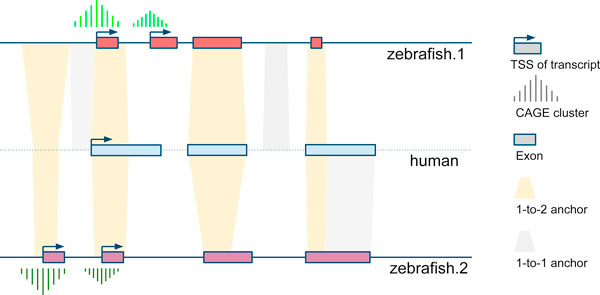
**The principle of comparing homologous genes using alignment anchors**.

To align two homologous loci, we used UCSC chain and net alignment data [27], which is a whole genome alignment by blastz [28]. Any alignment block in the UCSC chain database is taken as an anchor to link two loci. If a region in the reference species aligns to only one locus in the target species, we denoted it as a 1-to-1 anchor; otherwise, we extracted the overlapping parts of *M *(two or more) anchors and defined as 1-to-*M *anchor. For those having many (*M *> 2) aligned loci (e.g. genes by tandem duplication or from a large protein family), we only took the two highest scoring ones and display them as 1-to-2 anchors. The 1-to-2 mammal:zebrafish orthologs originating from teleost whole-genome duplication are expected to have 1-to-2 anchors. To distinguish the anchors from different scenarios, we marked them in different colors (by default, 1-to-1 anchors in gray, and 1-to-2 in blue: see Figure 1).

For human:human homolog comparison, we used the UCSC selfChain alignment to generate the anchors for paralogous loci. If no selfChain anchors are found, a link to Ensembl clustalW alignment is given. For zebrafish:zebrafish, we used human:zebrafish 1-to-2 chain alignment as a bridge to get zebrafish:zebrafish alignment due to the absence of zebrafish selfChain data at present. This method cannot detect the region only conserved between two zebrafish loci, but not conserved in human, for example those fast evolving regions specific in human lineage [29]; on the other hand, it can provide insight into the probable ancestral state of the locus[6].

## Results and discussion

### Identification of homologous genes

We extracted an initial homolog set from Ensembl Compara [30,31]. Out of 21416 human protein-coding genes, 79% and 51% have orthologs in mouse and zebrafish respectively. There are 29721 human:human paralog pair combinations altogether. To investigate how many of them are duplicates from 1R/2R WGD we grouped the paralogs by their last common ancestor. As shown in Figure S1, the largest category, which includes ~8400 human duplicates, falls in the time span before the split between bony fish (e.g. zebrafish) and tetrapods, and after the split between lancelets and jawless fish. This corresponds well with the proposed 1R/2R WGD timing (see Figure S1 in Additional file 1).

**Figure 2:**
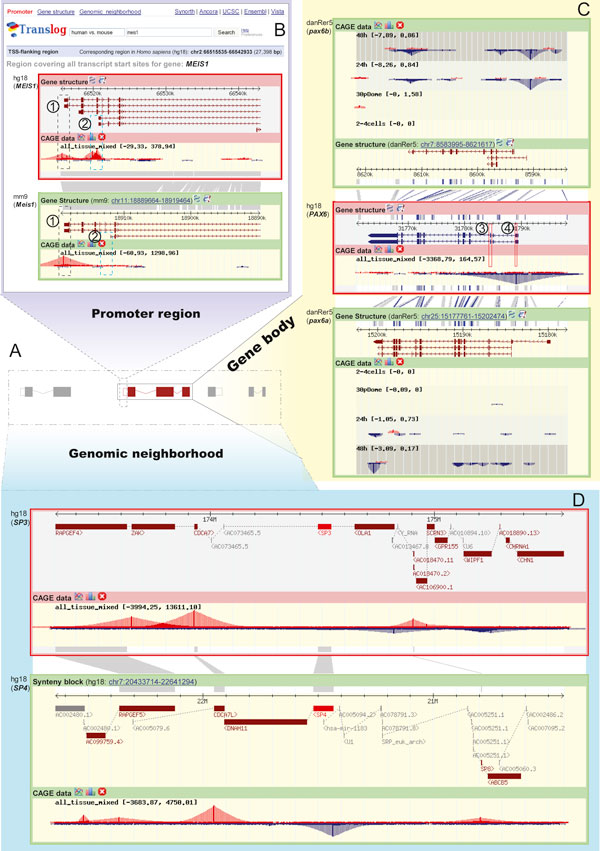
**Translog structure and three views modes**. A) Definition of three views in Translog. B) Promoter view of the *MEIS1 *(human) vs. *Meis1 *(mouse). C) Gene structure view, with *PAX6 *(human) vs. *pax6a/b *(zebrafish) comparison. D) Genomic neighborhood view, with *SP3 *(human) vs. *SP4 *(human) comparison.

Out of all human:zebrafish 1-to-2 orthologous genes, we wanted to determine how many date from teleost-specific WGD (3R WGD in Figure S1 in Additional file 1). To exclude the cases which have arisen by zebrafish-specific tandem duplications, ideally we should infer it from phylogenetic tree. A recent study [32], which identified gene duplicates retained from the last, teleost-specific WGD, found 615 human:zebrafish orthologs from the teleost WGD with high or medium confidence; most (94%) of them are included in the 1-to-2 orthologs we have defined here.

To study the expression divergence and differential promoter use of orthologous genes, we mapped CAGE tag clusters (TCs) to the human and mouse Ensembl genes. Most of the CAGE tags have a corresponding tissue in mouse and human in which they were detected. Only 7 out of 55 of those tissues in mouse do not have corresponding human tissue, whereas all the human tissues have corresponding mouse tissues (see Table S1 in Additional file 1). If multiple TCs map to one gene, the TC with the highest expression is chosen as representative TC. ~90% of the 1-to-1 orthologous gene pairs (13895 pairs in total) have at least one TC associated with them in both species.

### Comparing transcriptional initiation in homologous genes using Translog

Users can compare homologous genes and their CAGE data in three different views (Promoter, Gene structure, and Genomic neighborhood, see Figure 2A) through the links in the top-left corner of the Translog start page (see Figure 2B). The 'Promoter' view shows a region covering all transcription start sites (from both Ensembl transcripts and RefSeq genes) and extends 500 bp upstream and downstream (Figure 2B). The 'Gene structure' view shows the exon-intron structures of the pair of homologs (Figure 2C). The 'Genomic neighborhood' view shows the conservation of the query gene and its neighborhoods using the anchor of gene homology (Figure 2D). Translog currently supports comparison of human:human, human:mouse, human:zebrafish and zebrafish:zebrafish (not for zebrafish CAGE data right now). We aim to expand this list in the future to provide other perspectives or enable the study of other instances of whole-genome duplications, after the suitable genome assemblies and expression data become available. The first on the list are the new Zv8 zebrafish genome alignment data (whose annotation is still incomplete at present) and the upcoming zebrafish CAGE data, followed by the lamprey genome for studying the 2R whole genome duplication directly.

#### Basic usage

For any supported query identifier (Ensembl gene ID, HGNC gene symbol or gene synonyms), the browser shows CAGE data and corresponding genomic features relevant to the input query. In the right corner of each page there are links to the external resources (e.g. Synorth [33], Ancora [34], UCSC Browser [27], Ensembl [30] and Vista[35]) for each displayed region.

##### Promoter view

We define a (proximal) promoter region as a 1000 bp region centering on the TSS of a transcript. A genomic region spanned by the union of all promoter regions for the query gene is displayed in the reference genome, same for the target genome. Alignment anchors (if any) are also displayed linking the two loci, which can assist the user in mapping the homology of transcription start sites. This is particularly useful if a gene has several transcripts with different TSSes. For example, in Figure 2B, the human gene *MEIS1 *has 6 transcription isoforms with four different TSSes while its mouse ortholog *Meis1 *has 3 transcripts with two different TSSes. Most of the TSSes are covered by CAGE tag cluster, with different peak heights (corresponding to tissue-weighted expression level).

After we align the transcripts of the two genes by the alignment anchors (the gray bar between the red frame and green frame in Figure 2B), we can inspect the sharing and turnover of TSSes between the homologous transcripts. For example, the leftmost transcripts (the black dotted frame ① in Figure 2B) of the two *Meis1 *genes apparently share a CAGE cluster, indicating a shared ancestry of this particular promoter. On the other hand, the transcript with strongest expression (② in Figure 2B) in human *MEIS1 *does not have a CAGE cluster in the same position in its orthologous transcript in mouse. Looking at the difference in peak heights of each CAGE cluster, we can spot cases in which the most highly expressed transcript in one gene is not always the most expressed one in its ortholog. Compared to the methods used by previous studies (e.g. [14]), Translog can be used to define pairs of homologous transcripts more precisely.

##### Gene structure view

In this view, we define a transcript region as a region containing a transcript and a 500 bp flanking region both upstream and downstream of it. Analogously to the Promoter view, a region spanning the union of all transcripts for the query gene will be displayed for both loci, along with the anchors connecting them. By linking two homologous genes with alignment anchors, this view can be used to distinguish the structural heterogeneity of the coding region and pinpoint major differences in intron-exon structure and splice form usage between related genes. Figure 2C shows human *PAX6 *locus along with its two zebrafish not all human *PAX6 *exons are conserved in zebrafish; the 4th exon of *ENST00000379123 *is not conserved at all, while its second exon is only conserved in zebrafish *pax6a*.

After assigning the CAGE cluster to its transcript, the user can also investigate the relationship of expression changes and exon structure heterogeneity between homologous genes. Park et al.[14] classified each pair of duplicate genes into one of two structural categories: completely similar and incompletely similar. The latter were further classified in one of the three non-overlapping groups: 5' similar, 3' similar, and neither 5' nor 3' similar, with different extent of expression correlation. Using the 'Gene structure' view in Translog, the study of these kinds of correlations can be enhanced by quantifying the exon structure similarity only for those transcript pairs with shared TSS, instead of classifying them into a limited number of categories.

##### Genomic neighborhood view

This view displays the gene contents and CAGE data in a wider region around the query gene (see Methods). For human:zebrafish, we used the synteny blocks from[36]. For comparisons whose split events are too close (e.g. human:mouse ortholog from ~80 Myr ago) or too far (e.g. human:human paralogs from 1R/2R WGD ~550 Myr ago), we used a 2 Mb region centering on the query gene and its homologs. This view can be used to detect the synteny blocks dating from ancient segmental or whole genome duplications. For example, three genes in the human *SP3 *gene locus (*RAPGEF4*, *CDCA7 *and *AC018470.2 *[synonym of *SP9*]) also have paralogs next to *SP4 *(*RAPGEF5*, *CDCA7L *and *SP8*, respectively; see Figure 2D), with conserved gene order and orientation. This indicates that *SP3 *and its paralogous gene *SP4 *are not a consequence of the SP gene family expansion, but rather from duplications of whole loci, most likely whole genome duplications. Moreover, we found that the neighborhood synteny for *SP3 *is also conserved in another paralogous locus in which the arrangement of ZAK, *SP3 *and *SP9 *is mirrored by their paralogs *ZPK*, *SP1 *and *SP7*, respectively (Figure S2 in Additional file 1). This suggests that *SP3 *and *SP1 *neighborhoods arose from another duplication event, distinct of that that separated the ancestral neighborhoods of SP3 and SP4. But which duplication event occurred first? A previous study [37] found that *SP1 *and *SP3 *are more closely related to each other, and that their common ancestor was split from the ancestral form of SP4 by an earlier duplications. However, according to the picture (Figure S2 in Additional file 1) that we get from the synteny data, a more parsimonious explanation is that the gene content of *SP1 *and *SP4 *neighborhoods are from the result of a complementary gene loss after a recent duplication, while *SP3 *locus is an out-group to them.

## Conclusion

Translog is designed for studying the gene expression divergence of homologous genes across vertebrate genomes or paralogous loci within a genome. Based on the homology and CAGE expression data available for human, mouse and zebrafish, it provides a genome browser for visualizing and assessing the difference between homologous genes, on three different levels: promoter usage, gene structure changes, and genomic neighborhood conservation. One of the novel features of Translog is the possibility to display the comparison of two genomic loci in one browser by using alignment anchors. CAGE data is used to identify the true transcription start sites, measure the expression strength, and define the turnover or shift of promoter usage between homologous features. We anticipate that Translog will be highly useful for examining the factors that influence expression divergence between homologous genes.

## List of abbreviations used

WGD: whole genome duplication; GRB: genomic regulatory block; HCNE: highly conserved non-coding element; TSS: transcription start site; CAGE: cap analysis gene expression; bp: base pair; Myr: million years;

## Competing interests

The authors declare that they have no competing interests.

## Authors' contributions

XD and BL designed the study. XD analyzed the data set, designed the Translog web resource and the underlying database, and generated examples and figures for the manuscript. AA prepared the CAGE data set and analyzed the data set. XD, AA, YS and BL wrote the manuscript.

## Supplementary Material

Additional file 1 **Supplementary Table S1 and Figures S1, S2**. A document file contains supplementary Table S1 and Figures S1, S2. Click here for file
