# Supplementary material for: Translog, a web browser for studying the expression divergence of homologous genes
Source: BMC Bioinformatics. 2010 Jan 18;11(Suppl 1):S59. doi: 10.1186/1471-2105-11-S1-S59 (PMC3009532; doi:10.1186/1471-2105-11-S1-S59)
Supplement: Additional file 1 — Supplementary Table S1 and Figures S1, S2. A document file contains supplementary Table S1 and Figures S1, S2. [file 1471-2105-11-S1-S59-S1.pdf]

Supplementary

Table

Table S1. Percentage of 1-to-1 human-mouse orthologous having tag clusters around their promoter regions.
86.2 % of the both orthologous gene-pairs are covered by at least 1 CAGE tag from all libraries. Similar percentage for Lung libraries is 57.6 % and for Liver libraries is 52.2 %.

	% of covered 1-to-1 orthologs in mouse by CAGE tag clusters (TCs)		% of covered 1-to-1 orthologs in human by CAGE tag clusters (TCs)	
	tag tresholds for TCs >0	tag tresholds for TCs >=25	tag tresholds for TCs >0	tag tresholds for TCs >=25
All libraries	90.4 %	67.2 %	92.8 %	69.9 %
Lung libraries	73.4 %	26.3 %	63.6 %	10.2 %
Liver Libraries	60.8 %	18.6 %	65.9 %	17.3 %

Figures

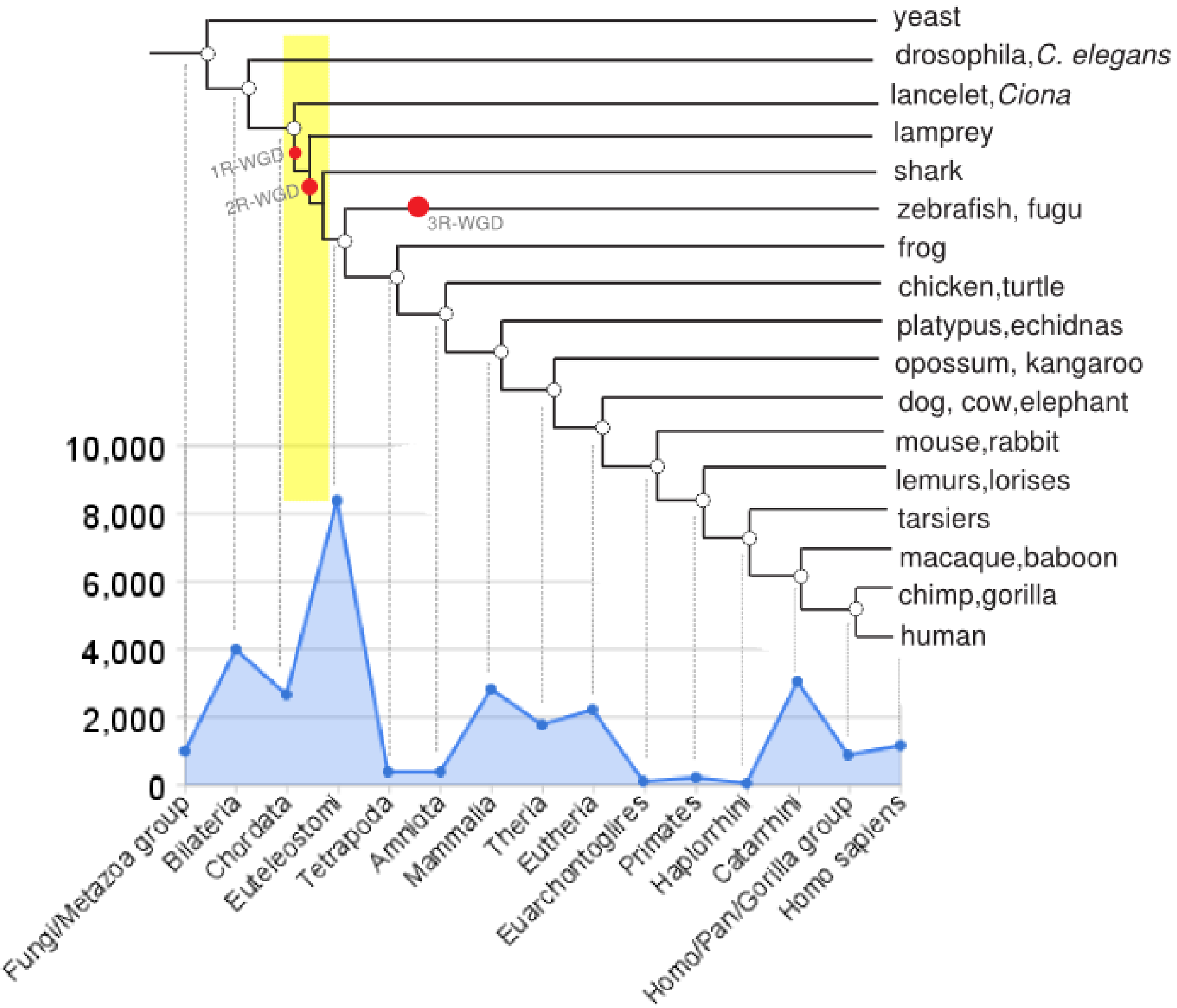

Figure S1. Evolution timing and statistics of last common ancestors of human paralogs. The tree shows representative species that have sequenced genomes. The red dots mark the whole genome duplication events. The yellow shading is the time period during which 1R and

2R WGD happened. The lower half shows counts of human:human paralogs categorized according to their last common ancestor. The data for rounds of WGD is from [38] and [39].

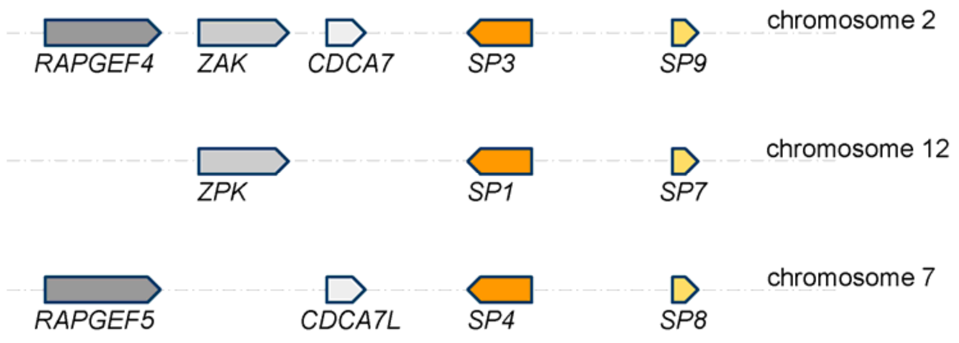

Figure S2. Simplified genomic positional relationship of human SP gene family and their conserved neighborhoods.
The figure shows members of human SP family and their genomic neighborhood. Genes of the same color are paralogs.
